# A novel pyroptosis-related prognostic signature for lung adenocarcinoma: Identification and multi-angle verification

**DOI:** 10.3389/fgene.2023.1160915

**Published:** 2023-04-03

**Authors:** Xinyue Wang, Jing Zhou, Zhaona Li, Xiuqiong Chen, Qianhui Wei, Kaidi Chen, Richeng Jiang

**Affiliations:** ^1^ Tianjin Medical University Cancer Institute and Hospital, National Clinical Research Center for Cancer, Tianjin, China; ^2^ Key Laboratory of Cancer Prevention and Therapy, Tianjin’s Clinical Research Center for Cancer, Tianjin, China; ^3^ Department of Thoracic Oncology, Tianjin Lung Cancer Center, Tianjin Cancer Institute and Hospital, Tianjin Medical University, Tianjin, China

**Keywords:** lung adenocarcinoma, risk signature, immunotherapy, immune checkpoint, pyroptosis, prognosis

## Abstract

**Background:** Lung adenocarcinoma (LUAD) is an aggressive disease of heterogeneous characteristics with poor prognosis and high mortality. Pyroptosis, a newly uncovered type of programmed cell death with an inflammatory nature, has been determined to hold substantial importance in the progression of tumors. Despite this, the knowledge about pyroptosis-related genes (PRGs) in LUAD is limited. This study aimed to develop and validate a prognostic signature for LUAD based on PRGs.

**Methods:** In this research, gene expression information from The Cancer Genome Atlas (TCGA) served as the training cohort and data from Gene Expression Omnibus (GEO) was utilized as the validation cohort. PRGs list was taken from the Molecular Signatures Database (MSigDB) and previous studies. Univariate Cox regression and Lasso analysis were then conducted to identify prognostic PRGs and develop a LUAD prognostic signature. The Kaplan-Meier method, univariate and multivariate Cox regression models were employed to assess the independent prognostic value and forecasting accuracy of the pyroptosis-related prognostic signature. The correlation between prognostic signature and immune infiltrating was analyzed to examine the role in tumor diagnosis and immunotherapy. Further, RNA-seq as well as quantitative real-time polymerase chain reaction (qRT-PCR) analysis in separate data sets was applied in order to validate the potential biomarkers for LUAD.

**Results:** A novel prognostic signature based on 8 PRGs (BAK1, CHMP2A, CYCS, IL1A, CASP9, NLRC4, NLRP1, and NOD1) was established to predict the survival of LUAD. The prognostic signature proved to be an independent prognostic factor of LUAD with satisfactory sensitivity and specificity in the training and validation sets. High-risk scores subgroups in the prognostic signature were significantly associated with advanced tumor stage, poor prognosis, less immune cell infiltration, and immune function deficiency. RNA sequencing and qRT-PCR analysis confirmed that the expression of CHMP2A and NLRC4 could be used as biomarkers for LUAD.

**Conclusion:** We have successfully developed a prognostic signature consisting of eight PRGs that providing a novel perspective on predicting prognosis, assessing infiltration levels of tumor immune cells, and determining the outcomes of immunotherapy for LUAD.

## 1 Introduction

Lung cancer is a leading cause to cancer-associated mortality globally, with a 5-year overall survival rate of approximately 15% (Molina et al., 2008; Schabath and Cote, 2019). Non-small cell lung cancer (NSCLC) constitutes 75–80% of lung cancer cases, with lung adenocarcinoma (LUAD) being the most prevalent subtype of NSCLC (Herbst et al., 2018). Advanced NSCLC is associated with a poor prognosis and a low worldwide 5-year survival rate (Chen et al., 2014). In recent years, the emergence of tumor immunotherapy has completely changed the traditional treatment mode, resulting in therapeutic benefits for some patients (Steven et al., 2016; Suresh et al., 2018; Yang et al., 2019). In spite of this, it is worth noting that immunotherapy is not an effective treatment strategy for all cancer patients. In fact, immune checkpoint inhibitors have demonstrated a limited clinical effectiveness in treating 85% of cancer patients, primarily due to inherent or acquired resistance to these therapies(Xiao et al., 2020). The underlying mechanisms contributing to this resistance may have been attributed to the tumor microenvironment (Wu and Dai, 2017).

In the era of cancer immunotherapy, all cancers in human can be classified into three phenotypes based on their anti-tumor immune response status: immune-inflamed, immune-excluded, and immune-desert. Tumors with high expression levels of PD-L1 and significant immune cell infiltration are referred to as “hot tumors” and are more responsive to immunotherapy. On the other hand, “cold tumors” or “immune-excluded” and “immune-desert” phenotypes, have little to no immune cell infiltration and show poor results with immunotherapy(Chen and Mellman, 2017).

Pyroptosis is a type of regulated cell death (RCD) that is triggered by the formation of plasma membrane pores caused by members of the gasdermin protein family, usually as a result of inflammatory caspase activation(Galluzzi et al., 2018). Different inflammasomes trigger caspases-1 upon infective and immunological challenges. The pyroptosis executive protein, gasdermin D (GSDMD) and gasdermin E (GSDME), the proteins responsible for executing pyroptosis, are downstream of inflammatory caspase proteases(Shi et al., 2015; Sborgi et al., 2016). Inflammatory caspase proteases cleave GSDMD to release the gasdermin-N terminal domain, which recognizes and binds phospholipid molecules on the cell membrane, forms membrane pores, leading to cell rupture and pyroptosis(Ding et al., 2016; Liu et al., 2016; Liu and Lieberman, 2017). Research has indicated that pyroptosis may play a role in inhibiting the development of cancer. Downregulation of GSDMD in gastric cancer has been linked to its development and progression (Wang et al., 2018). Conversely, overexpression of GSDMD has been associated with larger tumor sizes, later TNM stages, and poorer survival rates in LUAD (Gao et al., 2018). Unlike GSDMD, several studies have implicated GSDME as a tumor suppressor(Galluzzi et al., 2018; Rogers et al., 2019). Chemotherapy drugs can cause pyroptosis by caspase-3 cleavage of GSDME in NCI-H522 lung cancer cells(Wang et al., 2017). Similarly, cisplatin and paclitaxel induce pyroptosis in A549 cell line through activation of caspase-3/GSDME(Zhang et al., 2019). The results of these studies suggest that pyroptosis may have potential as a cancer diagnostic marker and therapeutic target. Recent studies have also shown that pyroptosis mediated by gasdermin is a cytotoxic lymphocyte killing mechanism that enhances anti-tumor immunity (Zhou et al., 2020). Pyrophosis occurs in a small number of tumor cells, which can effectively regulate tumor immune microenvironment and enhance anti-tumor immune response(Wang et al., 2020). As a tumor suppressor, GSDME activates pyroptosis and enhances anti-tumor immunity. Increased expression of GSDME leads to more active tumor-infiltrating natural killer and CD8^+^ T lymphocytes, as well as increased phagocytosis of tumor cells by tumor-associated macrophages(Tang et al., 2020). These results provide a strong theoretical basis for further research on pyroptosis and cancer. Targeting pyroptosis is expected to develop new immunotherapy drugs, which can cooperate with immune checkpoint inhibitors to play an anti-tumor role and improve the strength of immunotherapy effect.

In recent years, multiple gene-based prognostic signatures have been extensively investigated and utilized to forecast the prognosis of various types of tumors, public databases are being analyzed to identify prognostic signatures related to pyroptosis in tumors. Ju et al. demonstrated that a pyroptosis-related signature could predict survival in skin cutaneous melanoma patients(Ju et al., 2021). Ye et al. identified a risk gene signature for ovarian cancer that was associated with pyroptosis(Ye et al., 2021). Another study detected a pyroptotic-related signature that was capable of predicting infiltration of the immune microenvironment in gastric cancer and its prognosis (Shao et al., 2021). However, additional validation is needed to support the clinical use of the Pyroptosis-related models. In this study, we aimed to provide a more robust verification of a pyroptosis-related genes (PRGs) signature for predicting survival and immunotherapy response in lung adenocarcinoma by leveraging multiple datasets and analytical approaches.

## 2 Materials and methods

### 2.1 Data acquisition and preparation

The differentially expressed PRGs between normal and tumor tissues were determined using gene expression data from the Genotype-Tissue Expression (GTEx) and The Cancer Genome Atlas (TCGA) databases obtained from UCSC Xena data hubs (https://xena.ucsc.edu/). The RNA sequencing (RNA-seq) expression data of TCGA-LUAD, along with corresponding clinical information such as gender, age, tumor size, tumor stage, survival time and survival status, were used as the training set. After excluding samples with incomplete clinical information, 497 TCGA-LUAD samples were analyzed (Supplementary Table S1). Before further analysis, the expression data were transformed into transcripts per kilobase million (TPM) values. To validate the gene signature, we selected the GSE72094 dataset from the GEO database (https://www.ncbi.nlm.nih.gov/geo/).

### 2.2 PRGs and functional enrichment analysis

The Molecular Signatures Database (MSigDB, https://www.gsea-msigdb.org/gsea/msigdb website) and previously published articles were used to gather 51 genes related to pyroptosis (Supplementary Table S2). The “clusterProfiler” package was applied to analyze Gene Ontology (GO) and Kyoto Encyclopedia of Genes and Genomes pathway (KEGG) pathway data to examine the potential molecular mechanisms of differences(Yu et al., 2012). *p*-value <0.05 was considered as statistically significant.

### 2.3 Construction and validation of the prognostic signature

The prognostic value of the PRGs was determined by performing univariate Cox regression analysis. Genes with a *p*-value of less than 0.05 were considered statistically significant and further included in the least absolute shrinkage and selection operator (LASSO) regression analysis. LASSO regression, which was carried out using the “glmnet” R package, is effective in addressing the issue of multicollinearity in regression analysis (Friedman et al., 2010; Simon et al., 2011). The prognostic signature was established by selecting the candidate genes at the minimum value of the penalty parameter λ), resulting in an optimal signature. A risk score was calculated for each patient using the formula below:

$$risk\;score=\sum_{i=1}^{n}\left[\left(expGene\right)i\times coefi\right]$$

(expGene represents the gene expression value, coef represents the regression coefficient, and n represents the total number of genes). Each TCGA-LUAD patient was assigned a risk score based on the formula above. In accordance with the median risk score value, patients were divided into high-risk and low-risk groups.The difference in overall survival (OS) between the two groups was analyzed using Kaplan Meier survival curves, and a two-sided log-rank test was performed to determine the ability of the subgroups to differentiate patient outcomes in LUAD. Additionally, a time-independent receiver operating characteristic (timeROC) curve was performed to evaluate the predictive accuracy of the prognostic signature.

### 2.4 Development of a nomogram

In order to assess whether the signature was associated with prognostic factors and clinical characteristics, such as tumor stage, gender, and age, we conducted a univariate and multivariate Cox regression analysis. A prognostic signature that showed significant differences in both univariate and multivariate Cox analyses was considered to be an independent risk factor. These independent risk factors were then used to develop a nomogram, which was used to predict the one-, three-, and 5-year survival probability of LUAD patients.

### 2.5 Immune correlation analysis

With single-sample gene set enrichment analysis (ssGSEA) in the “gsva” R package, the activity of immune-related pathways and the infiltrating score of immune cells were calculated to verify the immune correlation of prognostic signature(Hänzelmann et al., 2013). Besides, the relationship between prognostic signature and immune-activity-related signatures and immune-checkpoint-related signatures was additionally analyzed using the Spearman coefficient and Wilcoxon rank-sum.

### 2.6 Protein expression profile of PRGs

The Human Protein Atlas (HPA) database (https://www.proteinatlas.org/) was used to identify the protein expression of immunohistochemical staining of PRGs in patients with LUAD.

### 2.7 Human specimens and cell lines

The Cancer Biobank of Tianjin Medical University Cancer Institute and Hospital provided all the tissue specimens for the study, including 25 cancer tissues and their paired normal adjacent tissues (ANTs) for RNA-seq analysis and 16 cancer tissues and paired ANTs for qRT-PCR analysis. The access to these tissue specimens was granted by the institutional Ethics Committee (bc2022263) and all cases were confirmed to be lung adenocarcinoma (LUAD) through pathology. The patients provided informed consent.

For cell line experiments, human LUAD cell lines A549 and H1299 were acquired from the American Type Culture Collection, while human LUAD cell line H1395 and a human normal bronchial epithelium cell line BEAS-2B were purchased from the Xiehe cell bank in Beijing, China. All cell lines were grown in RPMI-1640 medium (Gibco Laboratories, Grand Island, NY, United States) with 10% fetal bovine serum under 37°C and 5% CO_2_ conditions in a humid atmosphere.

### 2.8 RNA extraction and RNA-seq

Total RNA from fresh frozen tissues was extracted with Trizol (#9109, TaKaRa, Tokyo, Japan) according to the manufacturer’s recommendations. The cDNA library construction and transcriptome sequencing were carried out by Geneseeq (Nanjing, China). The sequencing libraries were created using the KAPA Stranded RNA-Seq Kit (KAPA, United States) with index codes to identify each sample. The libraries were combined and paired-end sequencing with 2 × 150 bp reads was performed using the NovaSeq6000 PE150 platform. The base calling was done using bcl2fastq v2.19.0.316 (Illumina, Inc.) to generate sequence reads in FASTQ format (Illumina 1.8 + encoding). Quality control was performed using Trimmomatic (version 0.36). Gene-level quantification was carried out with RSEM (version 1.2.31) and differential expression analysis was conducted using the R package DESeq2.

### 2.9 Real-time qRT-PCR analysis

After RNA extraction, cDNA was obtained through RNA reverse transcription using the StarScript III All-in-one RT Mix with gDNA remover kit (#A230-10, GenStar, Beijing China) with a 20 μL system (37°C, 2 min; 50°C, 15 min; 85°C, 2 min). The resulting cDNA was used for qRT-PCR with the SYBR Green PCR Kit (#A304-10, GenStar, Beijing China) on an ABI QuantStudio 5 (Q5) system (Applied Biosystems, United States). The qRT-PCR cycling profile consisted of 95°C/5s and 60°C/34s for 50 cycles. The internal control was glyceraldehyde 3-phosphate dehydrogenase (GADPH) and relative expression was calculated using the 2^−ΔΔCt^ method. Each PCR reaction was performed with three biological replicates and three technical repeats. The sequences of the relevant primers were.CHMP2A: 5′- CGC​GAG​CGA​CAG​AAA​CTA​GAG-3′5′- CCCGCATCAATACAAACTTGC-3′NLRC4: 5′-TACACAGCAGGACGAAGACTCAG-3′5′-GGCTTCCACAGATGACCCACA-3′GAPDH: 5′-GCA​CCG​TCA​AGG​CTG​AGA​AC-3′5′-ATG​GTG​GTG​AAG​ACG​CCA​GT-3′

### 2.10 Statistical analysis

**FIGURE 1:**
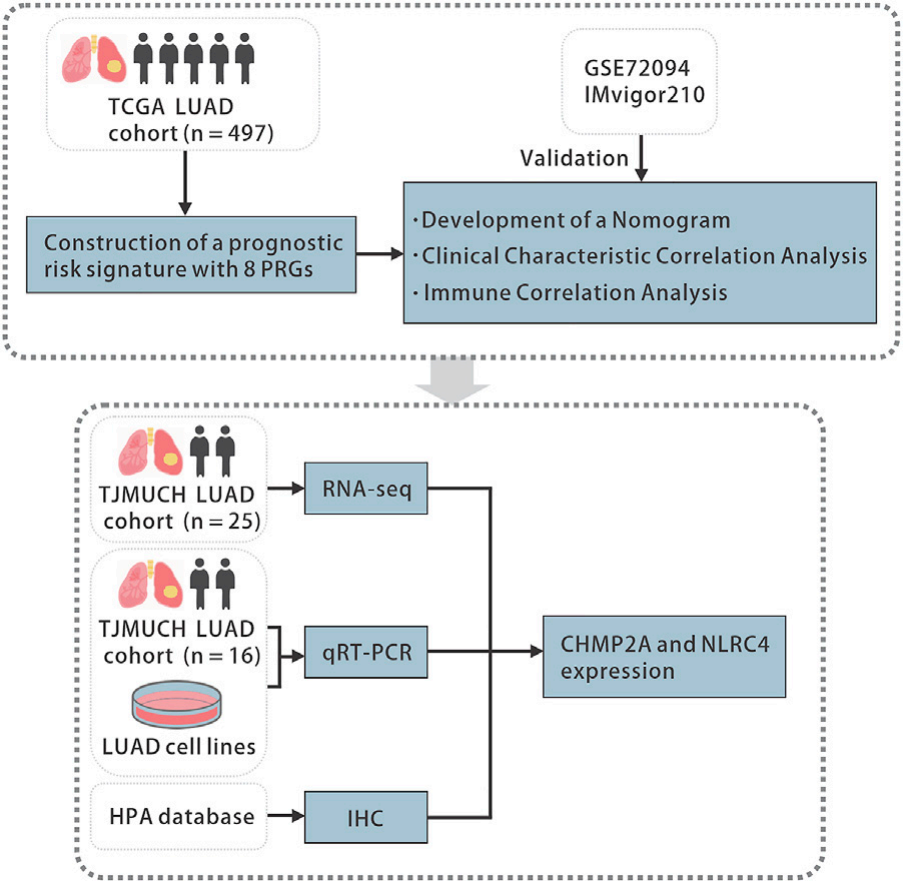
The flowchart of the study. TCGA, the Cancer Genome Atlas, LUAD, lung adenocarcinoma, PRGs, pyroptosis-related genes, qRT-PCR, quantitative real-time polymerase chain reaction, HPA, the Human Protein Atlas, IHC, immunohistochemistry.

The Wilcoxon rank sum test was used to assess the difference in PRGs expression between LUAD and normal tissues. The two-sided log-rank test was employed to compare the survival time between subgroups. To compare the difference between subgroups and clinical characteristics and immune infiltration levels, Student's t-test or the Wilcoxon rank sum test was utilized. Additional statistical methods used in the analysis are described in detail above. Results with *p*-value <0.05 was considered as statistically significant. Statistical significance was indicated in the figures as follows: ns *p*-value >0.05, * *p*-value <0.05, ** *p*-value <0.01, *** *p*-value <0.001, **** *p*-value <0.0001. All statistical analyses were accomplished with R software (version 4.2.1, R Foundation). The overall flowchart is shown in Figure 1.

## 3 Results

### 3.1 Identification of differentially expressed PRGs and functional enrichment analysis

**FIGURE 2:**
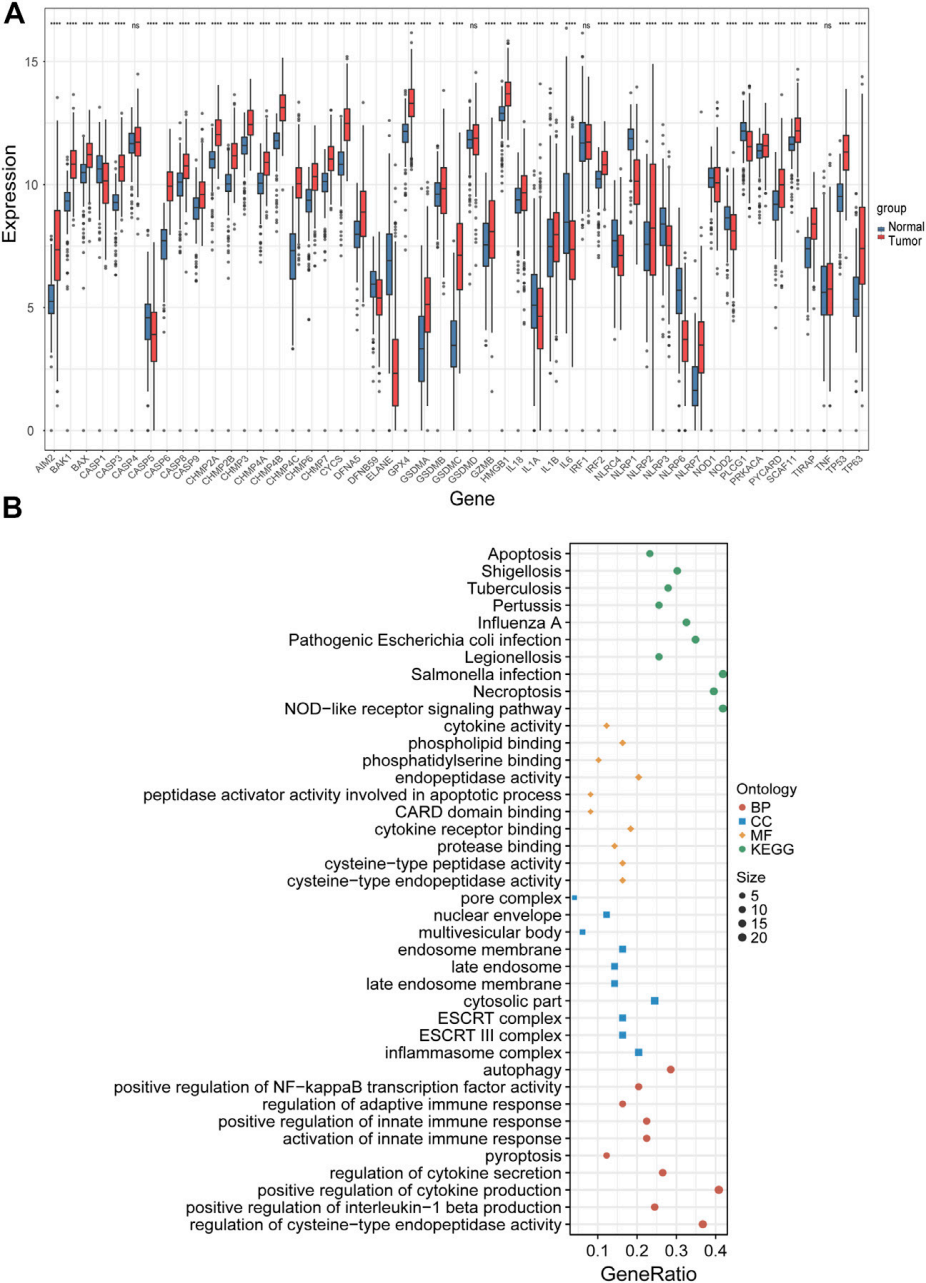
Identification of differentially expressed PRGs and functional enrichment analysis. **(A)** The expression levels of 51 PRGs between normal and tumor tissues. **(B)** The main results of the functional enrichment analysis of differentially expressed PRGs. BP, biological process, CC, cellular component, MF, molecular function, and KEGG, Kyoto Encyclopedia of Genes and Genomes.ns *p*-value >0.05, ** *p*-value <0.01, *** *p*-value <0.001, and **** *p*-value <0.0001.

The expression levels of 51 PRGs were compared between normal and tumor tissues, and 47 genes were found to be differentially expressed. Among them, the expression of DFNA5, TP53, TP63, BAX, BAK1, CASP8, CHMP2A, CHMP2B, CHMP3, CHMP4A, CHMP4B, CHMP4C, CHMP6, CHMP7, CYCS, GSDMA, GSDMB, GSDMC, AIM2, GPX4, PRKACA, IL18, IL1B, TIRAP, PYCARD, CASP9, CASP3, CASP6, CASP8, IRF2, SCAF11, HMGB1, NLRP2, NLRP7 was increased, while the expression of IL1A, IL1A, IL6, NLRP3, DFNB59, ELANE, NOD2, NOD1, CASP1, NLRC4, NLRP1, NLRP6, PLCG1 was decreased in LUAD compared with normal tissues (Figure 2A). To clarify the molecular function and pathway of PRGs, functional enrichment analysis was performed for 47 differentially expressed genes, which mainly enriched in positive regulation of cytokine production, pyroptosis, regulation of innate and adaptive immune response, positive regulation of cytokine production involved in immune response, positive regulation of NF-κB transcription factor activity, autophagy and pyroptosis, *etc.* (Figure 2B).

### 3.2 Construction of a prognostic signature in the TCGA cohort

**FIGURE 3:**
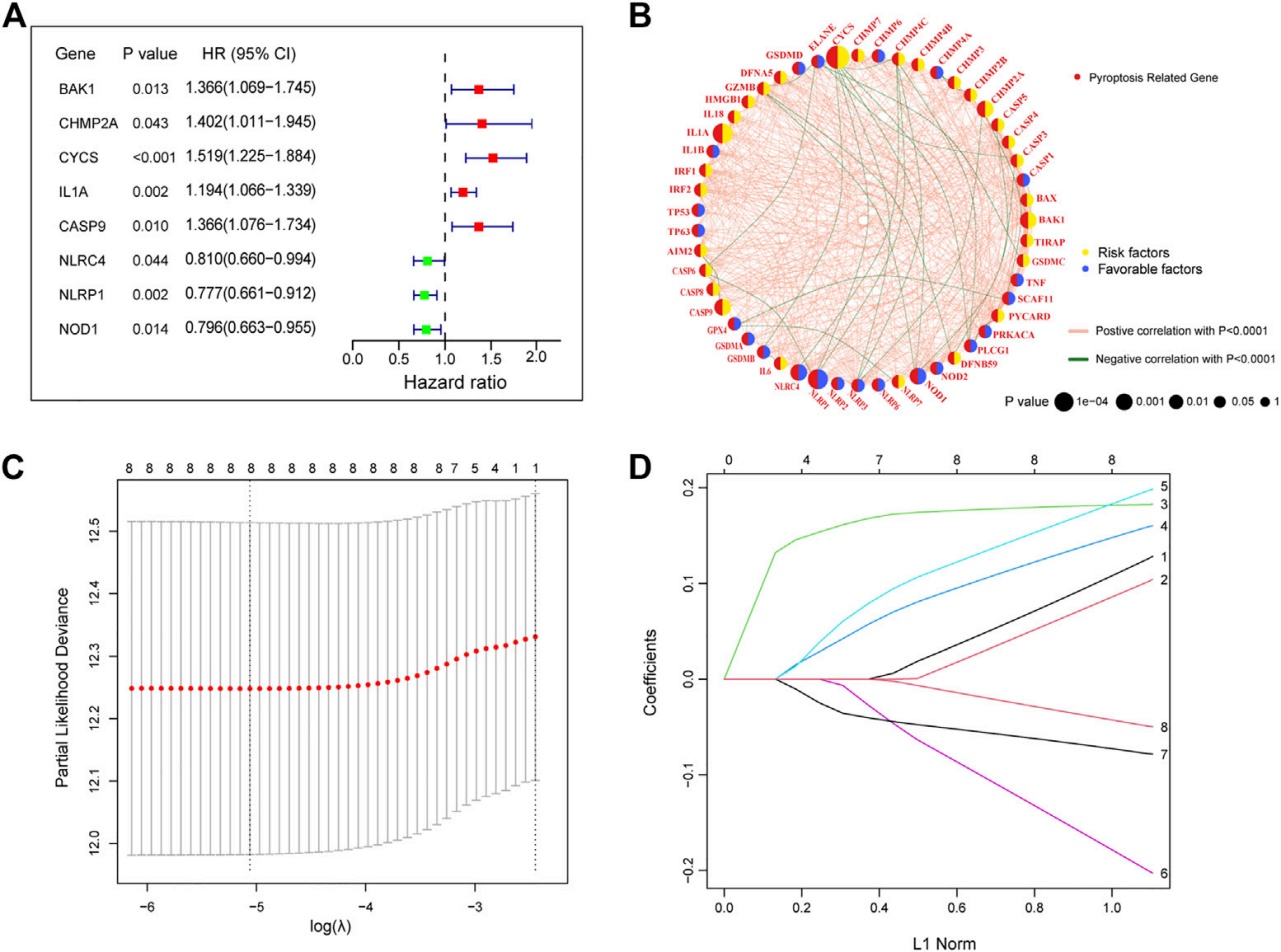
Construction of a prognostic signature in the training cohort **(A)** The forest plots of univariate Cox regression analysis. **(B)** Molecular interaction network map of PRGs. **(C)** LASSO coefficient profiles of the 8 PRGs. **(D)** Select the optimal value of λ by LASSO regression. LASSO, the least absolute shrinkage and selection operator.

**FIGURE 4:**
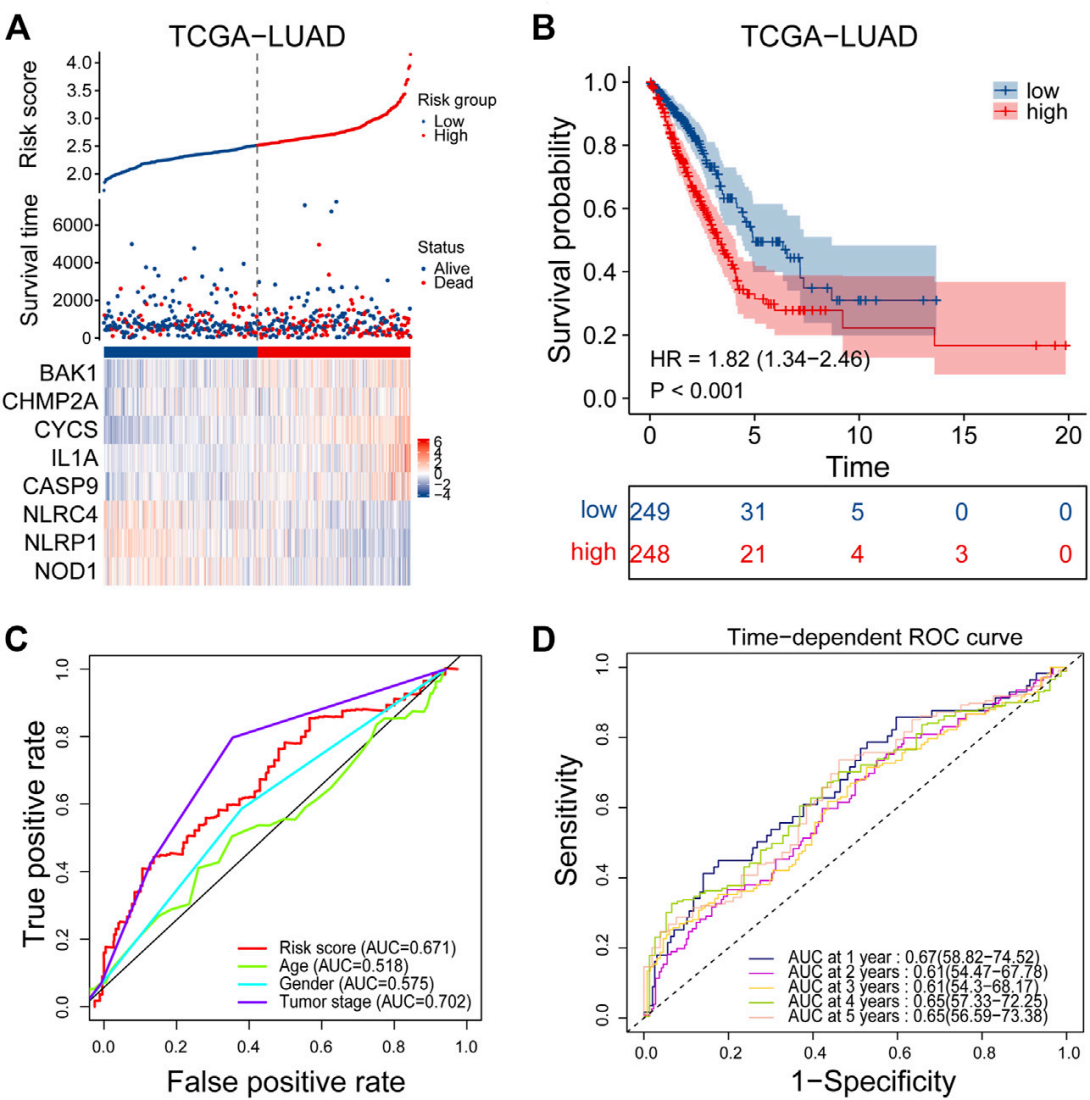
Performance evaluation of prognostic signature in the training cohort. **(A)** Heatmap of the curve of risk score, survival status and expression profiles of prognostic signature genes in high-risk and low-risk subgroup in the training cohort. **(B)** Kaplan-Meier survival analysis of the prognostic signature in the training cohort. **(C)** The AUC values of risk score and other clinical factors in the training cohort. **(D)** Time-dependent ROC analysis of the prognostic signature for predicting the 1-, 2-, 3-, 4- and 5- year overall survival in the training cohort. ROC, receiver operating characteristic; AUC, area under the ROC curve.

Univariate Cox regression analysis was performed on 47 differentially expressed PRGs, and 8 PRGs, BAK1, CHMP2A, CYCS, IL1A, CASP9, NLRC4, and NOD1, were found to be significantly correlated with the prognosis of LUAD (Figure 3A). The molecular regulatory network between PRGs is shown in Figure 3B. Further LASSO regression analysis was performed on the 8 PRGs to identify more suitable genes. The dotted line marked with the minimum value of logλ indicated that the corresponding gene was the best signature gene, and the LASSO regression coefficients were not zero (Figures 3C,D). Following stepwise regression analysis, BAK1(BCL2 Antagonist/Killer 1), CHMP2A (Charged Multivesicular Body Protein 2A, CYCS (Cytochrome C, Somatic), IL1A (Interleukin 1 Alpha), CASP9 (Caspase 9), NLRC4(NLR Family CARD Domain Containing 4), NLRP1 (NLR Family Pyrin Domain Containing 1) and NOD1 (Nucleotide Binding Oligomerization Domain Containing 1) were selected as candidate genes for constructing prognostic signature of this study. On the basis of the coefficients and expression, we developed a prognostic risk signature with 8 PRGs. The risk score = (0.115 × expression of BAK1) + (0.092 × expression of CHMP2A) + (0.182 × expression of CYCS) + (0.152 × expression of IL1A) + (0.188 × expression of CASP9) + (−0.187 × expression of NLRC4) + (−0.075 × expression of NLRP1) + (−0.045 × expression of NOD1). The Kaplan-Meier curve of 8 PRGs is shown in Supplementary Figure S1. Using the formula above, we calculated the risk score for each of the patients in the training set. The median risk score was used as a cut-off value to classify patients into high- and low-risk groups. Higher mortality rates were found in the high-risk score group, indicating that the high-risk score group may have a poorer prognosis. Heatmaps were used to visualize the expressions of 8 PRGs (Figure 4A). There were highly expressed transcripts of BAK1, CHMP2A, CYCS, IL1A, and CASP9 in the group with high-risk scores, whereas lowly expression was detected for NLRC4, NLRP1, and NOD1. Kaplan-Meier survival curves of the high- and low-risk groups in the TCGA training set were shown in Figure 4B, patients in high-risk groups had a shorter survival time (*p*-value <0.001). The ROC curve shows a comparison between the risk score and other independent clinical indices, the risk score (area under the ROC curve, AUC = 0.671) displayed a good performance among the indices (Figure 4C). The results of timeROC curve presented that 1-year AUC at 0.67, 2-year AUC at 0.61, 3-years at 0.61, 4-years at 0.65, and 5-years at 0.65 (Figure 4D). Based on the evaluation results above, the prognostic performance of the LUAD prognostic signature was not most perfect, and further validation was required.

### 3.3 Independent prognostic value of prognostic signature

**FIGURE 5:**
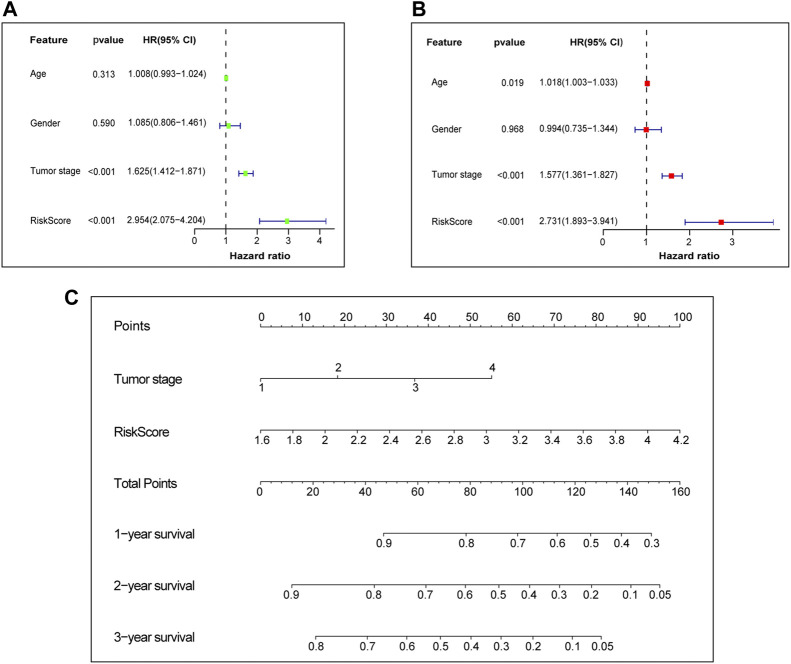
Independent prognostic value of prognostic signature. **(A)** Univariate Cox independent prognostic analysis of the TCGA cohort. **(B)** Multivariate Cox independent prognostic analysis of the TCGA cohort. **(C)** The nomogram to predict the 1-, 3- and 5-years overall survival of LUAD. TCGA, The Cancer Genome Atlas database. LUAD, lung adenocarcinoma.

Afterwards, Univariate and multivariate Cox regression analyses were performed to examine the independent prognostic value of risk score and other clinical features in the training set. Univariate Cox regression analysis found that risk score (Hazard ratio, HR = 2.954, *p*-value <0.001) and tumor stage (HR = 1.625, *p*-value <0.001) were unfavorable factors for survival time and significantly associated with OS (Figure 5A). We further conducted multivariate Cox regression analysis which suggested that the risk score may be an independent prognostic factor in predicting survival for LUAD patients in the training set (HR = 2.731, *p*-value <0.001, Figure 5B). The risk score and tumor stage are used as variables for building the nomogram, and the total score is calculated by adding the scores for each variable. This score can be used to estimate the one-, three-, and 5-year overall survival of patients with LUAD (Figure 5C). We further investigated the correlation between the prognostic signature and different clinical features. It was shown in Supplementary Figure S2 that the risk score was significantly correlated with age, lymph node metastasis, tumor stage, tumor response and survival status.

### 3.4 Validation of the prognostic signature in the GEO cohort

**FIGURE 6:**
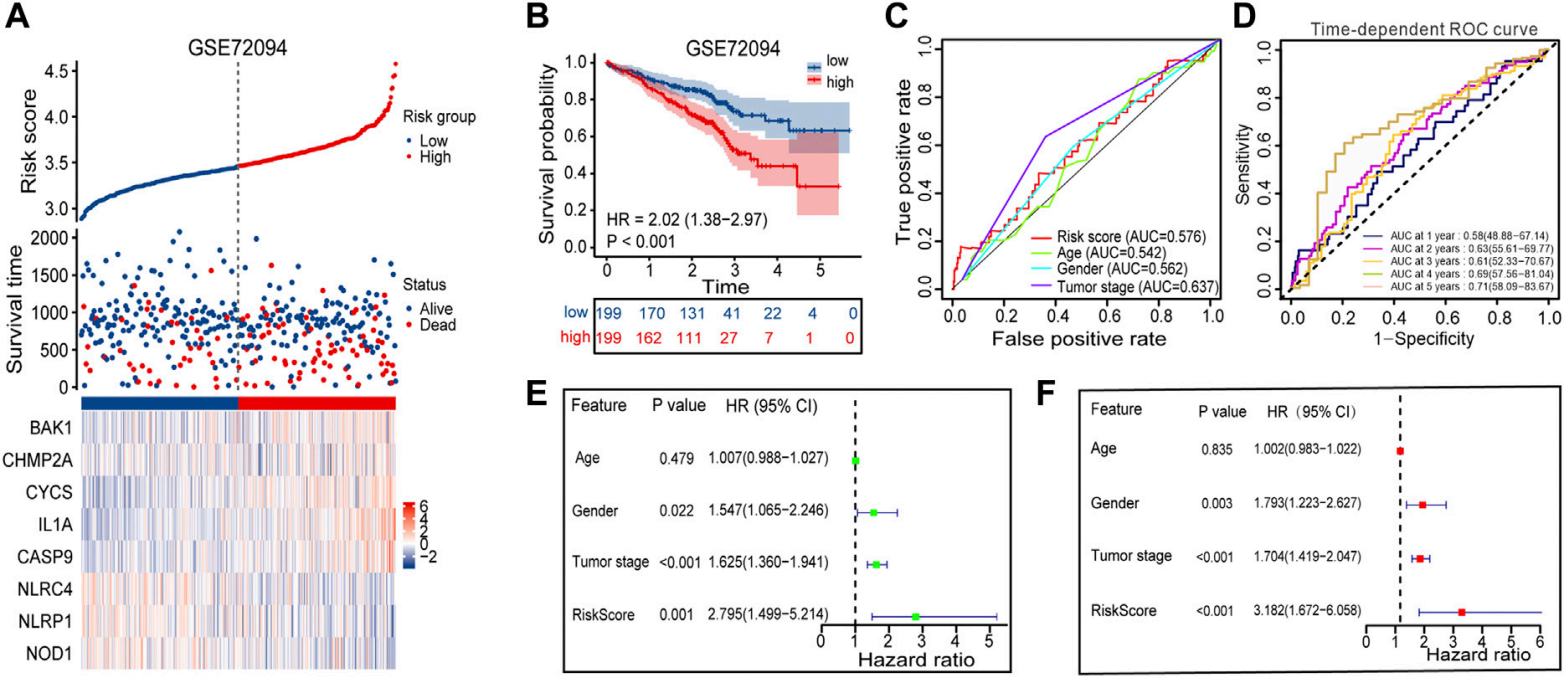
Performance evaluation of prognostic signature in the validation cohort. **(A)** Heatmap of the curve of risk score, survival status and expression profiles of prognostic signature genes in high-risk and low-risk subgroup in GSE72094 cohort. **(B)** Kaplan-Meier survival analysis of the prognostic signature in GSE72094 cohort. **(C)** The AUC values of risk score and other clinical factors in GSE72094 cohort. **(D)** Time-dependent ROC analysis of the prognostic signature for predicting the 1-, 2-, 3-, 4- and 5- year overall survival in GSE72094 cohort. **(E)** Univariate Cox independent prognostic analysis of the GSE72094 cohort. **(F)** Multivariate Cox independent prognostic analysis of the GSE72094 cohort. ROC, receiver operating characteristic; AUC, area under the ROC curve.

The GSE72094 dataset was chosen from the GEO database and used as external validation sets to verify the stability of the prognostic signature. A high proportion of patients in the high-risk group died compared to patients in the low-risk group, based on the distribution of gene expression profiles, risk score, and survival status from the validation set, which almost agree with the training set (Figure 6A). The Kaplan-Meier survival curves for GSE72094 (*p*-value <0.001) dataset confirmed that high-scoring patients had significantly poorer OS than low-scoring patients (Figure 6B). The predictive accuracy of prognostic signature was also validated in validation set. On the ROC curves in GSE72094, the AUC value about the risk score was 0.576 (Figure 6C). The AUC of timeROC curve in GSE72094 was 1-year is 0.58, 2-year is 0.63, 3-year is 0.61, 4-year is 0.69, and 5-year is 0.71 (Figure 6D). Univariate and multivariate Cox regression analyses were performed in the GSE72094 datasets to verify whether risk scores have an independent prognostic value in validation set. The verification results are consistent with those in the training set, the risk score also had independent prognostic value in GSE72094 data set (HR = 3.182, *p*-value <0.001, Figures 6E, F). According to the results of the validation set, the signature can be used for predicting the prognosis of LUAD with satisfactory sensitivity and specificity.

### 3.5 Mining the immune correlation of prognostic signature

**FIGURE 7:**
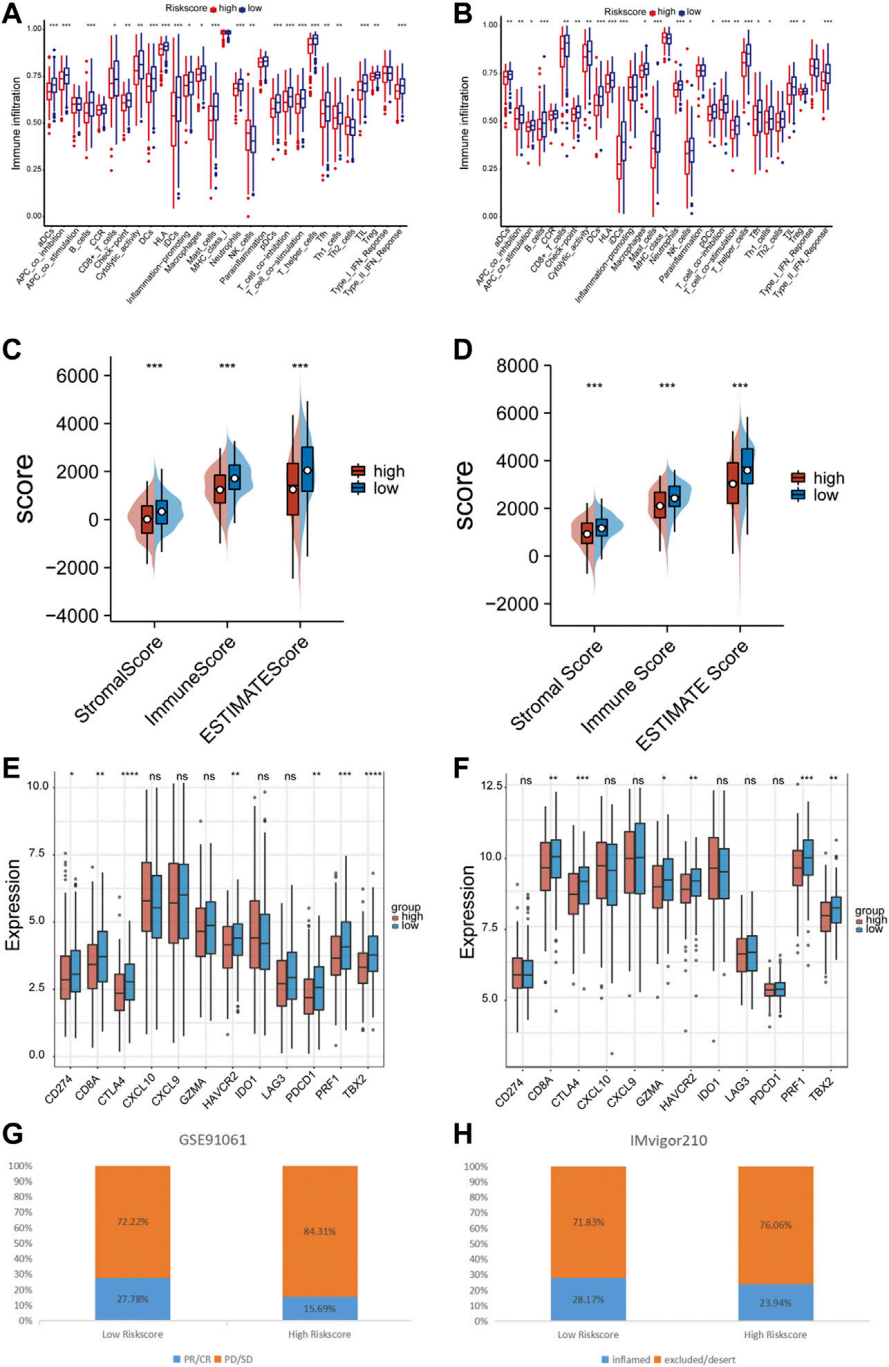
Immune correlation of prognostic signature. **(A)** The relationship between risk score and immune cells infiltration levels in the TCGA cohort. **(B)** The relationship between risk score and immune cells infiltration levels in the GSE72094 cohort. **(C)** The relationship between risk score and tumor immune microenvironment related scores in the TCGA cohort. **(D)** The relationship between risk score and tumor immune microenvironment related scores in the GSE72094 cohort. **(E)** The relationship between risk score and immune checkpoints expression levels in the TCGA cohort. **(F)** The relationship between risk score and immune checkpoints expression levels in the GSE72094 cohort. **(G)** The correlation between risk score and immune therapy response in the GSE91061 cohort. **(H)** The correlation between risk score and immune cell infiltration level in the IMvigor210 cohort. ns *p*-value >0.05, * *p*-value <0.05, ** *p*-value <0.01, *** *p*-value <0.001, and **** *p*-value <0.0001.

The ssGSEA method was employed to quantify the levels of enrichment for various immune cell subpopulations, associated functions, and pathways in both the TCGA and GEO cohorts, and to examine the correlation between the risk score and the level of immune cell infiltration. The high-risk subgroup typically showed lower levels of immune cell infiltration and a decrease in the activity of immune-related functions or pathways, when compared to the low-risk subgroup in the training set (Figure 7A). A similar finding was found in GSE72094 (except for the NK cells) (Figure 7B). The ESTIMATE score showed that patients in the low-risk group had higher TME scores in both data sets, indicating higher levels of stromal or immune cell infiltration (Figures 7C, D). We further selected 6 immune activity related genes, including CXCL9, CXCL10, CD8A, GZMA, TBX2 and PRF1, as immune-activity-related signature, and 6 immune checkpoints, including CTLA4, PDCD1, CD274, LAG3, IDO1 and HAVCR2, as immune-checkpoint-relevant signature. We observed that the high-risk subgroup was negatively correlated with the level of expression of these immune signature genes, especially with CD274, PDCD1 and CTLA4, the immune signature genes were significantly low expressed in the high-risk subgroup (Figures 7E, F). Patients in the GSE91061 cohort were divided into PR/CR and PD/SD groups based on their response to immunotherapy. The number of patients with PR/CR in the low-risk subgroup was higher than that in the high-risk subgroup (Figure 7G). According to the level of immune cell infiltration, patients in the IMvigor210 cohort were divided into two groups: Inflamed and Excluded/Desert. Patients in the low-risk subgroup had a majority of immunoinfiltrating cells (Figure 7H).

### 3.6 Validation of the expression level of CHMP2A and NLRC4 in LUAD tissues and cell lines

**FIGURE 8:**
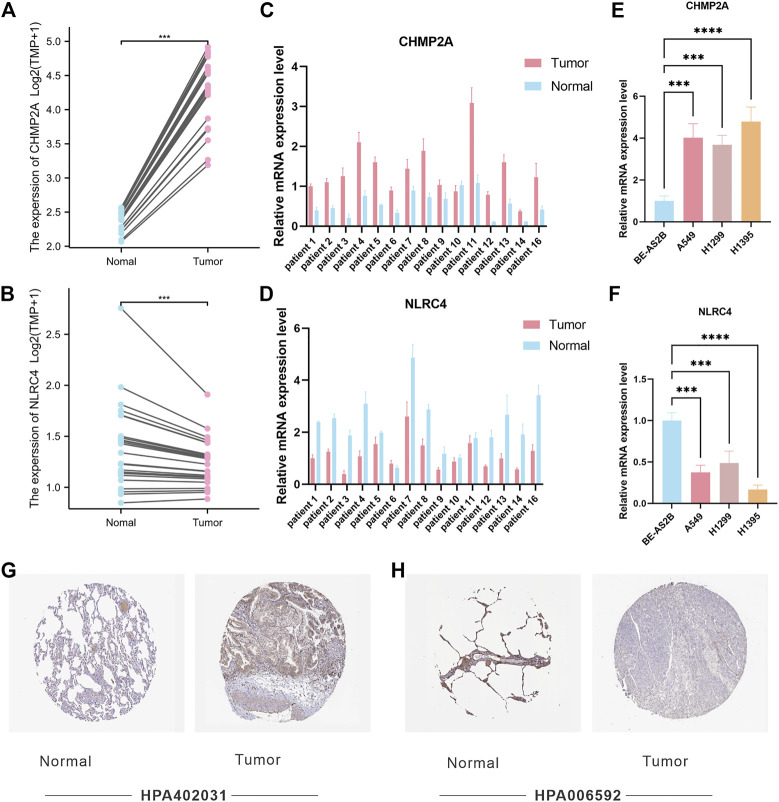
Validation of CHMP2A and NLRC4 expression. **(A, B)** The expression of CHMP2A and NLRC4 was evaluated in 25 LUAD tissues and paired ANTs. **(C, D)** Relative mRNA levels of CHMP2A and NLRC4 in 16 LUAD tissues and paired ANTs were verified *via* qRT-PCR. **(E, F)** Relative mRNA levels of CHMP2A and NLRC4 in LUAD cell lines and the normal bronchial epithelium cell line BEAS-2B were verified *via* qRT-PCR. **(G)** Protein expression of CHMP2A in the HPA database. **(H)** Protein expression of NLRC4 in the HPA database.

Among the PRGs, CHMP2A and NLRC4 showed the most significant difference in expression between cancer and adjacent cancerous normal tissues. It is believed that their functions exhibit promising potential in LUAD research. In order to verify the potential role of CHMP2A and NLRC4 in LUAD, we compared the expression levels of CHMP2A and NLRC4 in LUAD and normal tissues using a separate data set (n = 25) by RNA-seq. The results showed that the mRNA expression levels of CHMP2A in cancer tissues were lower than those in normal tissues, and the expression of NLRC4 was just the opposite (Figures 8A, B). Further, we validated the expression level of CHMP2A and NLRC4 in 16 cases of lung adenocarcinoma and matched adjacent cancerous tissues by using qRT-PCR analysis. The results showed that the CHMP2A mRNA expression levels in lung adenocarcinoma tissues were significantly higher than that in normal tissues (*p*-value <0.001), while the expression of NLRC4 in lung adenocarcinoma was significantly lower than that in normal tissues (*p*-value <0.001) which was consistent with our results above (Figures 8C, D). In addition, we also explored the mRNA expression of CHMP2A and NLRC4 in LUAD cell lines. As expected, the results showed that CHMP2A was highly expressed in LUAD cell lines while it was the opposite in NLRC4 (Figures 8E, F). By using the HPA database, we analyzed the protein expression levels of two genes in both LUAD and normal tissues. The expression of the CHMP2A protein was compared between LUAD and normal lung tissue using antibody HPA402031. Results showed that the expression of CASP1 protein was elevated in LUAD tissue compared to normal tissue (Figure 8G). Additionally, the expression of NLRC4 was compared between LUAD and normal lung tissue with the use of antibody HPA006592. It was found that the expression of NLRC4 was decreased in LUAD tissue compared to normal tissue (Figure 8H).

## 4 Discussion

In this study, we conducted a comprehensive analysis of TCGA and GEO databases and successfully constructed a pyroptosis-related prognostic signature that can assist in predicting the survival of LUAD patients. To identify PRGs with prognostic value, univariate Cox analysis and Lasso regression analysis were performed, and eight pyroptosis-related prognostic genes were identified to construct the pyroptosis-related prognostic signature of LUAD. The Kaplan-Meier survival curves showed significant worse survival for patients in the high-risk score subgroups. The sensitivity and specificity of the prognostic signature for predicting the prognosis of LUAD patients were moderately evident from the ROC curves. Univariate and multivariate Cox regression and nomogram analysis showed that the prognostic signature has potential independent prognostic value, which may assist TNM stage to predict prognosis. The above conclusions are also well validated in GSE72094 datasets.

CHMP2A and NLRC4 were found to be variables in the prognostic model, and the difference of their expression in cancer and adjacent cancerous normal tissues has been verified in separate cohorts from our hospital. Higher expression of CHMP2A and lower expression of NLRC4 were correlated with poor overall survival. Therefore, the PRG prognostic signature incorporating these two genes could be used to stratify LUAD patients into different risk groups and guide individualized treatment decisions. Previous literature identified that it has been proposed that CHMP2A, a subunit of endosomal sorting complexes required for transport III (ESCRT-III), may help enhance the effect of NK cells on tumor cells (Bernareggi et al., 2022). The data demonstrates that CHMP2A and extracellular vesicles released by tumors can induce programmed cell death in NK cells, thereby diminishing their capacity to efficiently eliminate cancer cells. NLRC4 inflammasome is known to play a protective role in tumorigenesis. In an azoxymethane and dextran sodium sulfate-induced colitis-associated colorectal cancer model, the NLRC4 inflammasome has a protective role in tumorigenesis (Hu et al., 2010). On the other hand, studies have revealed that it also suppresses tumor growth in an inflammasome-independent manner (Janowski et al., 2016). Specifically, NLRC4 is an important regulator of key inflammatory signaling pathways in macrophages, and is required for the development of protective CD4^+^ and CD8^+^ T cells that produce IFN-γ. Our results suggest that CHMP2A and NLRC4 could be promising targets in immunotherapy of LUAD, and the PRG signature incorporating CHMP2A and NLRC4 could be used to identify patients who are more likely to respond to immunotherapy. The further validation and refinement of the signature, as well as deeper investigation into the biological functions of CHMP2A and NLRC4, may lead to a better understanding of disease mechanisms and ultimately inform the development of new treatments and interventions.

The prognostic signature not only predicts the prognosis of LUAD patients but also shows a strong correlation with clinical features, immune cell infiltration, and immune-related functions and pathways. The high-risk score subgroup was found to be associated with advanced tumor stage and poor prognosis. Through functional enrichment analysis, it was discovered that the pyroptosis genes play a role in the regulation of immune responses. As we mentioned earlier, Wang et al. demonstrated that pyroptosis in only a small number of tumor cells is enough to trigger inflammatory response, regulates the tumor immune microenvironment, and turns on the powerful antitumor immune response of the T cells (Wang et al., 2020). Granzyme A is a type of serine protease that is produced by immune cells like cytotoxic T lymphocytes (CTLs) and NK cells. This substance can enter into tumor cells *via* perforin, which is found on the surface of the tumor cells. Once inside, Granzyme A specifically and effectively cuts the GSDMB protein, leading to the death of the tumor cells through a process known as pyroptosis (Zhou et al., 2020). In other words, cytotoxic lymphocyte-induced tumor cell death is pyroptosis. The activation of GSDME and caspase-3 through cleavage by Granzyme B leads to pyroptosis of tumor cells by NK cells, CD8^+^ killer lymphocytes, and chimeric antibody receptor T cells. This stimulates the anti-tumor immune response and impedes the growth of the tumor (Zhang et al., 2020). Pyroptosis does indeed turn ‘cold tumors’ into ‘hot tumors’, improving the effectiveness of immune checkpoint inhibitors. The high-risk subgroup was found to have lower levels of immune cell infiltration and decreased activity in immune functions or pathways. This points to a broader impairment of the immune system within this subgroup in the training cohort, and these findings were supported by the validation cohort analysis. As a result, it can be inferred that the poor survival outcomes experienced by the high-risk subgroup may stem from a reduced ability to combat the tumor. The number of immunoinfiltrating patients in the low-risk subgroup of the IMvigor210 cohort was higher than that in the high-risk group. The aforesaid data lead to the hypothesis that the poor survival outcome of patients in the high-risk category may be brought on by a reduction in anti-tumor immune activity. Furthermore, the high-risk subgroup showed a significant decrease in the expression of important immune checkpoint proteins, such as CD274, CTLA4, and PDCD1. This provides additional evidence that patients within this subgroup may not respond as well to immunotherapy. This hypothesis was confirmed in the GSE91061 cohort where more patients in the low-risk group achieved partial or complete responses than in the high-risk subgroup.

In recent years, numerous studies have focused on developing prognostic signatures related to pyroptosis to predict the prognosis of LUAD patients. Lin et al. conducted a comprehensive bioinformatics analysis and identified a predictive PRG signature comprising five genes(Lin et al., 2021). They further determined a regulatory axis that can predict the prognosis of LUAD patients. However, the prognostic signature was not validated in external datasets. Similarly, another study established a prognostic signature of five PRGs in LUAD and validated it in GEO datasets to explore the role of pyroptosis in LUAD (Gong et al., 2022). The validation experiments demonstrated that GPX4 and PRKACA were key elements of the prediction model. These studies suggest that many PRGs play vital roles in tumor occurrence and development, and their expression in tumor tissues has great potential to predict survival and immunotherapy efficacy. Since the role of pyroptosis in tumor development is just beginning to be understood, there are still many PRGs waiting to be discovered. With the discovery of more PRGs, the accuracy and specificity of the PRG model will further improve. Therefore, after constructing a prognostic signature, this study validated the prognostic signature using two external datasets and discovered the key roles of CHMP2A and NLRC4 through RNA sequencing data and qPCR validation. This not only improves the reliability of the model but also discovers new interaction-related molecular targets with promising prospects.

In conclusion, we have successfully developed a LUAD prognostic signature consisting of eight PRGs. This signature can be used to predict prognosis, assess tumor immune cell infiltration levels, and serve as a reliable tool for determining immunotherapy outcomes for LUAD. Our findings also offer valuable insights for the search of promising targets for immunotherapy of LUAD. However, it is important to note that this study has some limitations. Firstly, the gene signature was developed using publicly available databases, and we were unable to validate its prognostic value using data from our hospital due to the lack of complete clinicopathological and survival information. Further prospective studies will be needed to confirm the clinical utility of the pyroptosis-related prognostic signature. Moreover, further research is needed to explore the role of these genes in the prognosis of LUAD, including *in vitro* and *in vivo* experiments.

## Data Availability

The RNAseq data presented in the study are deposited in the SRA repository, accession number SRP422869 (https://www.ncbi.nlm.nih.gov/sra/?term=SRP422869).
