# Supplementary material for: A novel pyroptosis-related prognostic signature for lung adenocarcinoma: Identification and multi-angle verification
Source: Front Genet. 2023 Apr 3;14:1160915. doi: 10.3389/fgene.2023.1160915 (PMC10106613; doi:10.3389/fgene.2023.1160915)
Supplement: Supplementary file 2 [file Table1.DOCX]

# Supplementary Table 1 | Clinical characteristics of TCGA-LUAD patients.

| Characteristic | Levels | Overall |
| --- | --- | --- |
| n |  | 497 |
| Gender, n (%) | Female | 269 (54.1%) |
|  | Male | 228 (45.9%) |
| Tumor size, n (%) | T1 | 166 (33.4%) |
|  | T2 | 267 (53.7%) |
|  | T3 | 43 (8.7%) |
|  | T4 | 18 (3.6%) |
|  | TX | 3 (0.6%) |
| Lymph node metastasis, n (%) | N0 | 321 (64.6%) |
|  | N1 | 94 (18.9%) |
|  | N2 | 69 (13.9%) |
|  | N3 | 2 (0.4%) |
|  | NX | 11 (2.2%) |
| Distant metastasis, n (%) | M0 | 331 (66.6%) |
|  | M1 | 24 (4.8%) |
|  | MX | 142 (28.6%) |
| Tumor stage, n (%) | not reported | 7 (1.4%) |
|  | stage I | 267 (53.7%) |
|  | stage II | 118 (23.7%) |
|  | stage III | 80 (16.1%) |
|  | stage IV | 25 (5%) |
| Treatment response, n (%) | not reported | 166 (33.4%) |
|  | PD/SD | 123 (24.7%) |
|  | PR/CR | 208 (41.9%) |
| Survival state, n (%) | Alive | 317 (63.8%) |
|  | Dead | 180 (36.2%) |
| Age, median (IQR) |  | 66 (59, 72) |
| Survival time (Day), median (IQR) |  | 657 (422, 1126) |
