# Supplementary material for: A novel pyroptosis-related prognostic signature for lung adenocarcinoma: Identification and multi-angle verification
Source: Front Genet. 2023 Apr 3;14:1160915. doi: 10.3389/fgene.2023.1160915 (PMC10106613; doi:10.3389/fgene.2023.1160915)
Supplement: Supplementary file 3 [file DataSheet1.docx]

# Supplementary Figures


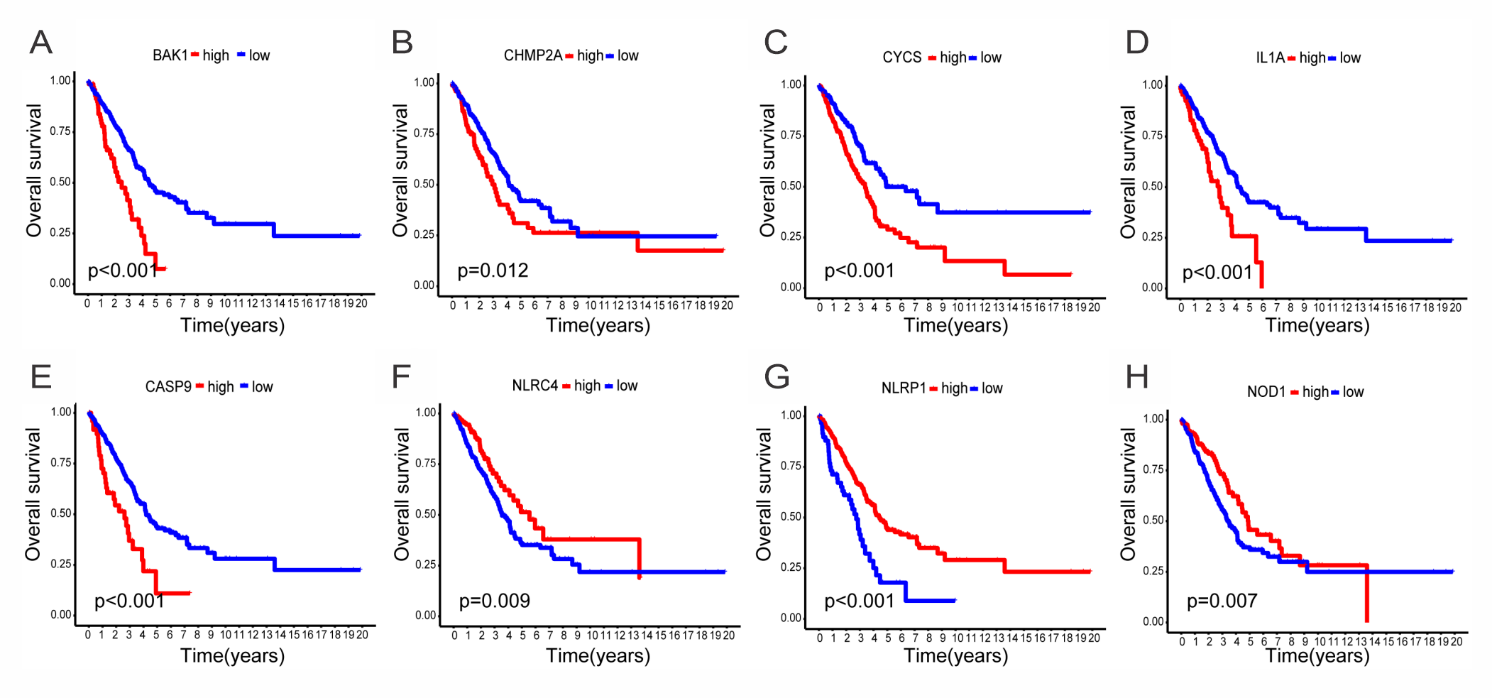


**Supplementary Figure S1 |** Kaplan-Meier survival analysis of the PRGs (A) survival curve of BAK1; (B) survival curve of CHMP2A; (C) survival curve of CYCS; (D) survival curve of IL1A; (E) survival curve of CASP9; (F) survival curve of NLRC4; (G) survival curve of NLRP1; (H) survival curve of NOD1.


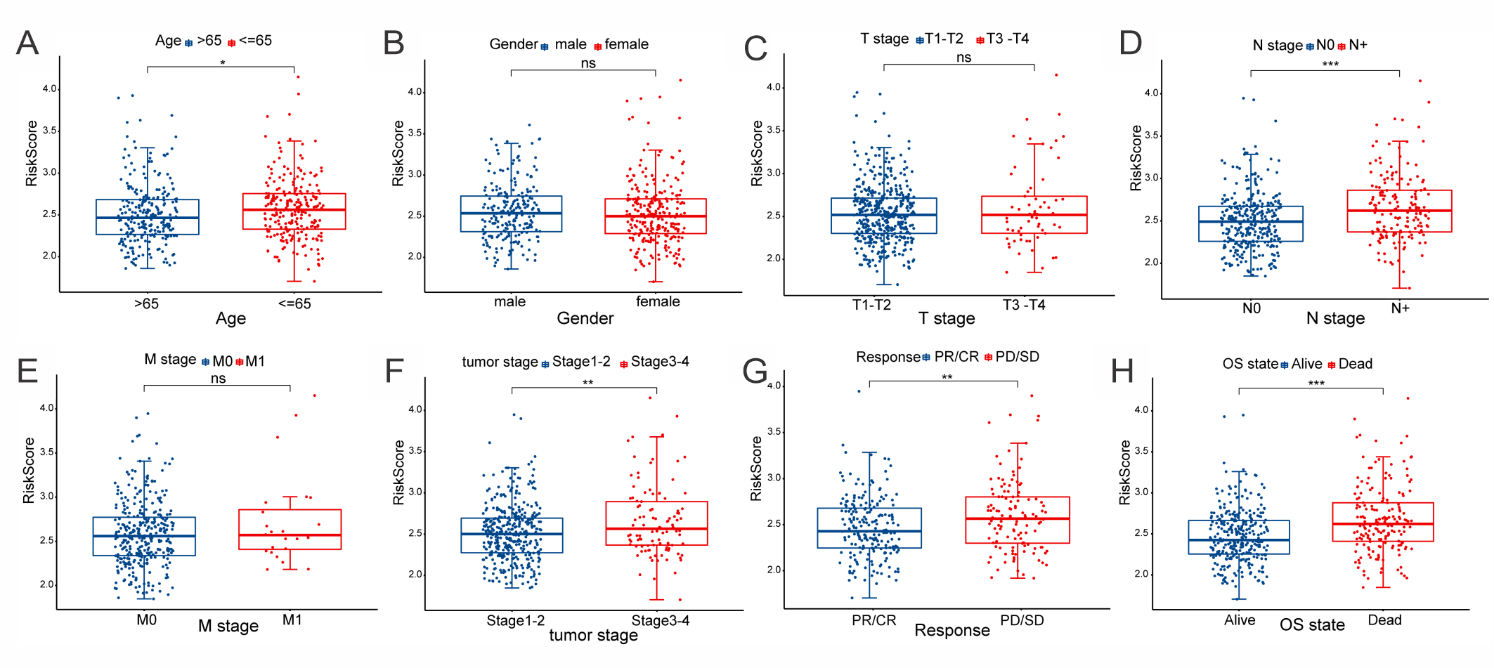


**Supplementary Figure S2 |** Prognostic signature and correlations with clinical characteristic. (A) the clinical correlation between risk score and age. (B) the correlation between risk score and gender. (C) the correlation between risk score and T stage. (D) the association between risk score and N stage. (E) the correlation between risk score and M stage. (F) the correlation between risk score and tumor stage. (G) the correlation between risk score and tumor response. (H) the correlation between risk score and survival status. ns *P*-value > 0.05, * *P*-value < 0.05, ** *P*-value < 0.01, and *** *P*-value < 0.001.
